# Tritium distribution in the ‘water-soil-air’ system in the Semipalatinsk Test Site

**DOI:** 10.1371/journal.pone.0297017

**Published:** 2024-04-04

**Authors:** Lyubov Timonova, Natalya Larionova, Almira Aidarkhanova, Oxana Lyakhova, Medet Aktayev, Zarina Serzhanova, Sergey Lukashenko, Vasiliy Polevik, Alexey Dashuk, Valeriy Monayenko, Sergey Subbotin, Assan Aidarkhanov

**Affiliations:** 1 Institute of Radiation Safety and Ecology, National Nuclear Center of the Republic of Kazakhstan, Kurchatov, Kazakhstan; 2 Shakarim State University of Semey, Semey, Kazakhstan; University of South Carolina, UNITED STATES

## Abstract

This article presents research findings on ^3^H in abiotic environmental compartments, specifically, the ‘water-soil-air’ system. All of the research areas are located within the Semipalatinsk Test Site (STS): the perimeter of the ‘Degelen’ site, the riverside zone of the Shagan river and the ‘background’ area–the southeastern part of the STS (SEP). As research progressed, numerical values of ^3^H and its species were revealed in various environmental compartments. The presence of ^3^H was registered not only in underground nuclear test locations but also in the ‘background’ area–SEP. Maximum ^3^H tritium concentrations in the water were detected at the ‘Degelen’ site (up to 57000±5000 Bq/kg) and the Shagan riv (up to 61500±6000 Bq/kg), in the air of the ‘Degelen’ site (up to 56±11 Bq/m^3^), in the soil of the ‘Degelen’ site (up to 5170±500 Bq/kg) and the Shagan riv (4100±400 Bq/kg) in the free water, at SEP (up to 1710±170 Bq/kg) in the organic constituent. Based upon all of the findings, ^3^H was found to be readily distributed in abiotic environmental compartments depending on certain conditions. Research suggests that water plays a key role in ^3^H migration processes in the natural system of interest. The second most but equally important constituent is soil and microorganisms of plant and animal origin living there. These assumptions are indirectly proven by research findings that show the HTO and HT air concentration dynamics depending on the sampling location.

## 1. Introduction

Due to its nuclear and physical characteristics, ^3^H, as a pure β-emitter, is one of the least dangerous isotopes from the perspective of external exposure [1]. Its half-life is T_1/2_ = 12.3 years, and its energy is 18.6 keV. However, because ^3^H is a hydrogen isotope that is incorporated in many organic compounds, it can be a source of human internal exposure through inhalation and ingestion. Due to the intake of ^3^H by β-emission, molecular structures and intermolecular bonds become disordered.

Under natural conditions, nuclear reactions with cosmic rays affecting atom nuclei of light chemical elements incorporated in the ambient air are a source of continued synthesis of ^3^H in the atmosphere. Among other compounds, tritium compounds such as ^3^H oxide (HTO), molecular hydrogen (HT) and methane (CH_3_T) are focused on [2].

The atomic industry, namely, nuclear power plants (NPP), currently accounts for the highest fate of releases and discharges of man-made ^3^H into the environment. ^3^H produced at NPPs, unlike other radionuclides, enters the environment by bypassing treatment facilities through liquid effluents in the form of tritiated water and gas releases because of its extremely high migration capacity [3]. As shown by predictive estimates, further development of nuclear energy will continue to favor the growth of ^3^H production and release into the environment [1].

Nuclear tests have been another source of a great deal of ^3^H produced in the environment. For instance, as a result of numerous tests conducted at the Semipalatinsk Test Site (STS), significant concentrations of ^3^H are currently also registered in environmental compartments. This radionuclide was detected close to locations of nuclear explosions in surface and ground waters [4, 5], air [6–10], plants [11–13], soil [14–18] and snow cover [19].

The article [1] quotes the analysis of data generalization on the behavior of ^3^H in different environmental components. Taking into account the relatively short half-life of ^3^H, its high migration capacity, and the availability of its several physical and chemical forms, modern knowledge about the regularities of ^3^H behavior in the environment is rather ambiguous. The migration of ^3^H in environmental components represents complex multiyear and multistage processes that depend on its spatial distribution, seasonal and interannual variability, weather conditions, and nonuniform distribution in the ecosystem. Accordingly, issues concerning the study of environmental ^3^H migration processes deserve special attention.

Also, from our point of view, it is worth highlighting separately a number of reactor experimental studies with tritium, in which special production of tritium is carried out to solve the problems of choosing materials for fusion reactor blankets. Such studies are carried out in various countries, including in Kazakhstan, at the IVG1.M (Kurchatov) and WWR-K (Almaty) research reactors [20–25].

Thus, the scale of tritium contamination of natural ecosystems located in the zone of influence of hazardous radiation facilities and nuclear installations may be underestimated. Once in the environment, tritium reacts with oxygen and is quickly integrated into numerous cycles of the biosphere as tritiated water (HTO). Due to its chemical properties, it is extremely mobile in biological systems and may be found in all hydrogenated molecules and associated water in the biosphere [1]. To correctly evaluate the content of ^3^H in the environment, it is important to understand the mechanisms of its distribution. At least three key components–water, soil and air–are the main link in the vital activities of all living things on earth and matter most in studying ^3^H migration.

Thus, the goal of the paper is to research the content and distribution of ^3^H in abiotic environmental compartments–water, soil and air.

All research was conducted at the STS. The major radioactive contamination status was generated by above ground and underground testing. The bulk of radionuclides, formed in aboveground explosions, has remained at the test epicenters, but a fraction, related to relatively small soil particles or molten rocks, were transported by the wind to considerable distances. More large-scale and complex processes affect the venues of underground nuclear testing and their surroundings, with transportation of radionuclides (including the ^3^H) by surface and underground water streamflows. Owing to the extensive distribution of ^3^H at individual STS spots, the test site is a unique ‘natural laboratory’ in which due to the presence of a great deal of ^3^H in the water, soil and air, one can conduct research on the distribution of ^3^H under natural conditions, which will allow the subsequent application of findings to other nuclear power facilities. At the same time, peculiarities in distribution of tritium in the ‘water-soil-air’ system at different sites of the STS may be different depending on the nature of radionuclide contamination and natural conditions.

## 2. Materials and techniques

### 2.1 Study area

**Fig 1:**
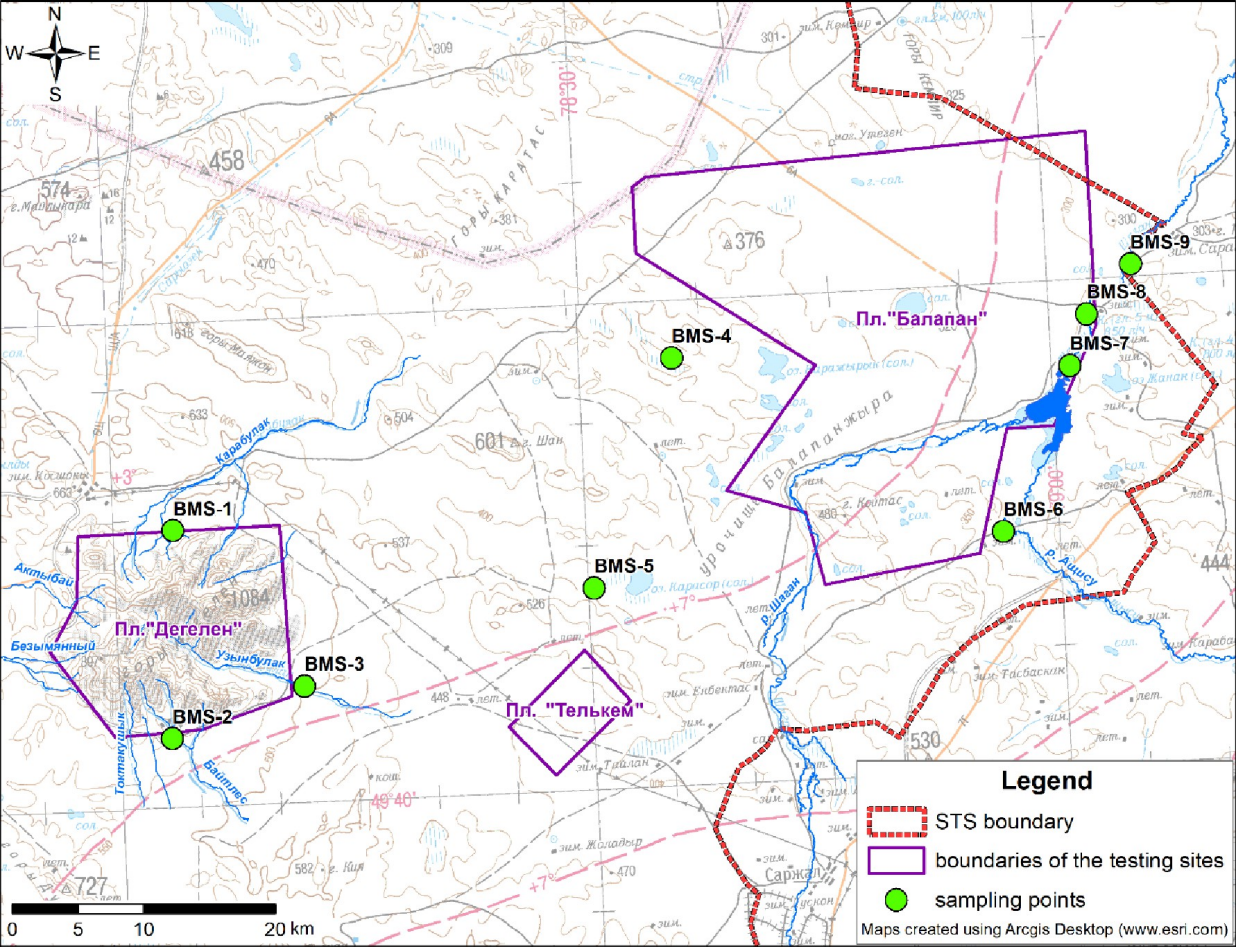
General schematic arrangement of BMS.

The STS territory with ^3^H being present in environmental compartments and represented both by various natural conditions and different sources of radioactive contamination was chosen for research. Nine spots were selected–so-called biological monitoring sites (BMSs) (Fig 1), at which soil, water and air were sampled.

Research spots were chosen on the border of the ‘Degelen’ site and in the riverside zone of the Shagan with the high content of ^3^H in environmental compartments of interest as well as in a conventionally ‘background’ area in the southeastern STS part (SEP), which contains ^3^H in environmental compartments at the level of background values.

The ‘Degelen’ site is located in the southern STS part. Underground nuclear tests were conducted here in mountain adits from 1961 through 1989. Their near-mouth spots contained a high content of ^3^H in the air [7, 26]. For research, tritium-contaminated water streams were selected–creeks that outflowed beyond the ‘Degelen’ site. The creeks are hydrologically linked to water streams flowing from adits, thereby causing the carry-over of ^3^H from nuclear test locations to adjacent areas. Values of ^3^H concentration in adit waters reach 340.000 Bq/kg [27]. Research areas were in the bed of three water streams: the Karabulak cr. (BMS-1), the Baitles cr. (BMS-2) and the Uzynbulak cr. (BMS-3) originating from adit 177 water stream. The location of the study areas is presented in the figure (Fig 1 in S1 Appendix).

Research spots on the ‘Degelen’ site are represented by soils of the meadow series that are thin (the soil thickness in the bed does not exceed 40–45 cm, the one in the riverside zone– 20–25 cm), fairly humous (up to 20%), and well-washed from readily soluble salts and carbonates. The bulk of plant biomass is concentrated in water stream flood plains represented by diverse meadow vegetation due to additional moistening. The projective cover of plants in the flood plain is 90%, and the productivity is 3–5 cwt/ha.

The Shagan riv. is located at the ‘Balapan’ site and is the most extended surface water stream flowing at STS along its eastern boundary. In 1965, at the confluence of the Shagan and Aschysu riv, an excavation fusion explosion was conducted for the purpose of creating an artificial reservoir, which resulted in the formation of the ‘Atomic Lake’ water body and an external reservoir. At a distance of 4.7 to 7.7 km from Shagan, maxima of ^3^H are registered in the water, reaching dozens and hundreds of thousands Bq/kg. Fracture waters are contaminated due to tritium in this section flowing along channels of tectonic disturbances [28, 29] from the ‘Balapan’ site area in which underground nuclear tests were conducted in boreholes.

**Fig 2:**
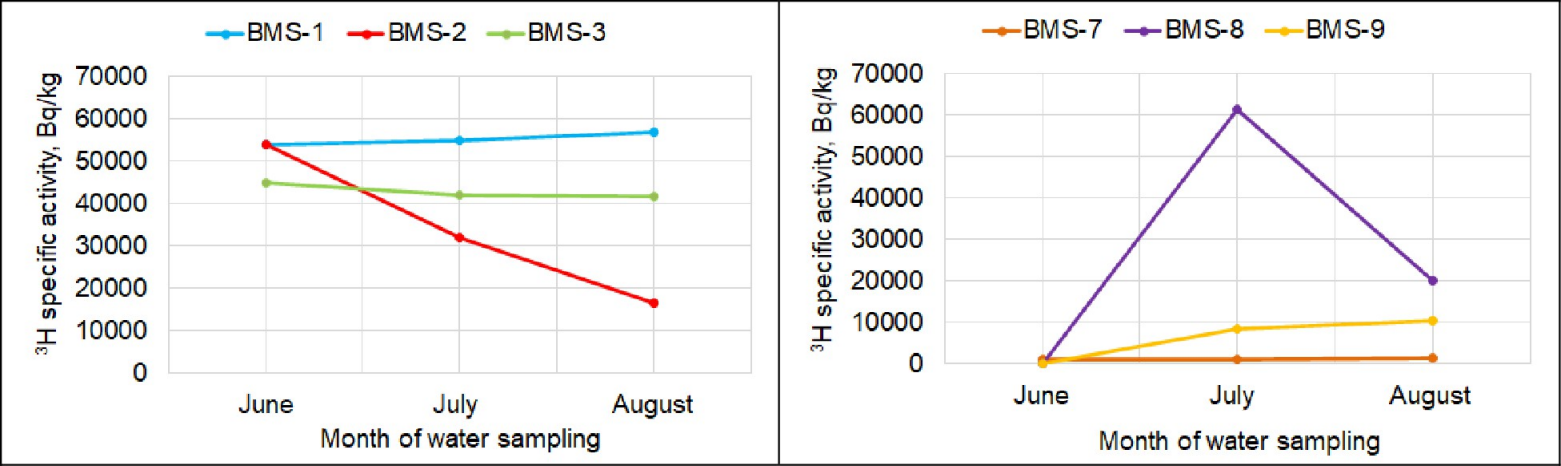
Dynamics of ^3^H concentration in the water of research sites. a) Dynamics of ^3^H concentration in the water of the ‘Degelen’ site, b) Dynamics of ^3^H concentration in the water of the riverside zone of the Shagan river.

^3^H distribution in environmental compartments of the riverside zone at the Shagan riv. was considered at 3 spots along the riverbed from ‘Atomic Lake’ and downstream. Selected sections were characterized by the maximum concentration of ^3^H in the water. That is, the BMS-7 spot (the vicinity of kilometer 2 away from the ‘Atomic Lake’, the left bank), the BMS-8 spot (the vicinity of kilometer 5 away from the ‘Atomic Lake’, the left bank) and the BMS-9 spot (the vicinity of the bed outlet beyond the ‘Balapan’ site, the right bank). Location of study areas in the riverside zone at the Shagan riv. is shown in the figure (Fig 2 in S1 Appendix).

Light chestnut soils are characteristic of the Shagan impact zone. The plant cover of the bed is built up by aquatic and aquatic-riverside species. The terrace is represented by steppe cenoses. The transitional scarp between the bed and the terrace showing plant succession is often covered in ruderal species.

A site in the STS SEP was chosen as a background area at a distance from nuclear test locations. This area is located between the ‘Degelen’ site in the west and the ‘Balapan’ site in the northeast. No nuclear tests were conducted in this territory. The radiological situation in SEP is attributed both to the global fallout and the fallout from aboveground nuclear tests conducted at the ‘Experimental Field’ and ‘Telkem’ [30]. The ^3^H concentration in this area is close to background values. However, low numerical values of ^3^H in the air and vegetation were registered in a number of places within the territory selected in the course of previous research [31].

**Fig 3:**
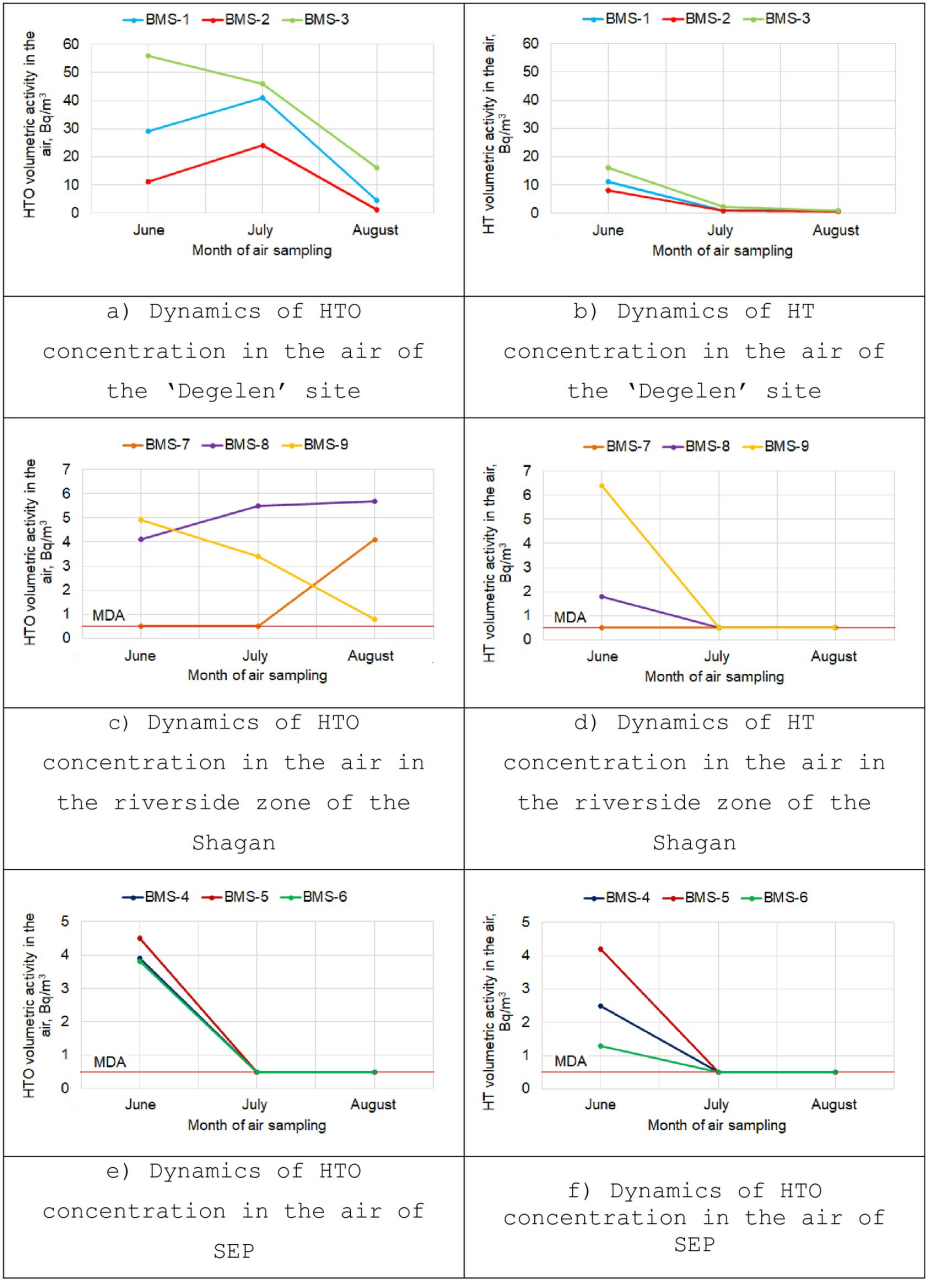
Dynamics of HTO and HT concentration in the air of research sites. a) Dynamics of HTO concentration in the air of the ‘Degelen’ site, b) Dynamics of HT concentration in the air of the ‘Degelen’ site, c) Dynamics of HTO concentration in the air in the riverside zone of the Shagan, d) Dynamics of HT concentration in the air in the riverside zone of the Shagan, e) Dynamics of HTO concentration in the air of SEP, f) Dynamics of HTO concentration in the air of SEP, MDA: minimum detectable activity.

In the southeastern STS part, research spots were near the ‘Balapan’ site (BMS-4, 5, 6) where water bodies are located (Fig 3 in S1 Appendix). The plant cover of SEP is reckoned among dry steppes on light chestnut soils and is characterized by the presence of saline soils. The vegetation is represented by a complex of gramineous-sandy needle grass-absinthial associations.

### 2.2 Environmental sampling

All soil, water and air sampling activities were carried out in the summertime.

**Fig 4:**
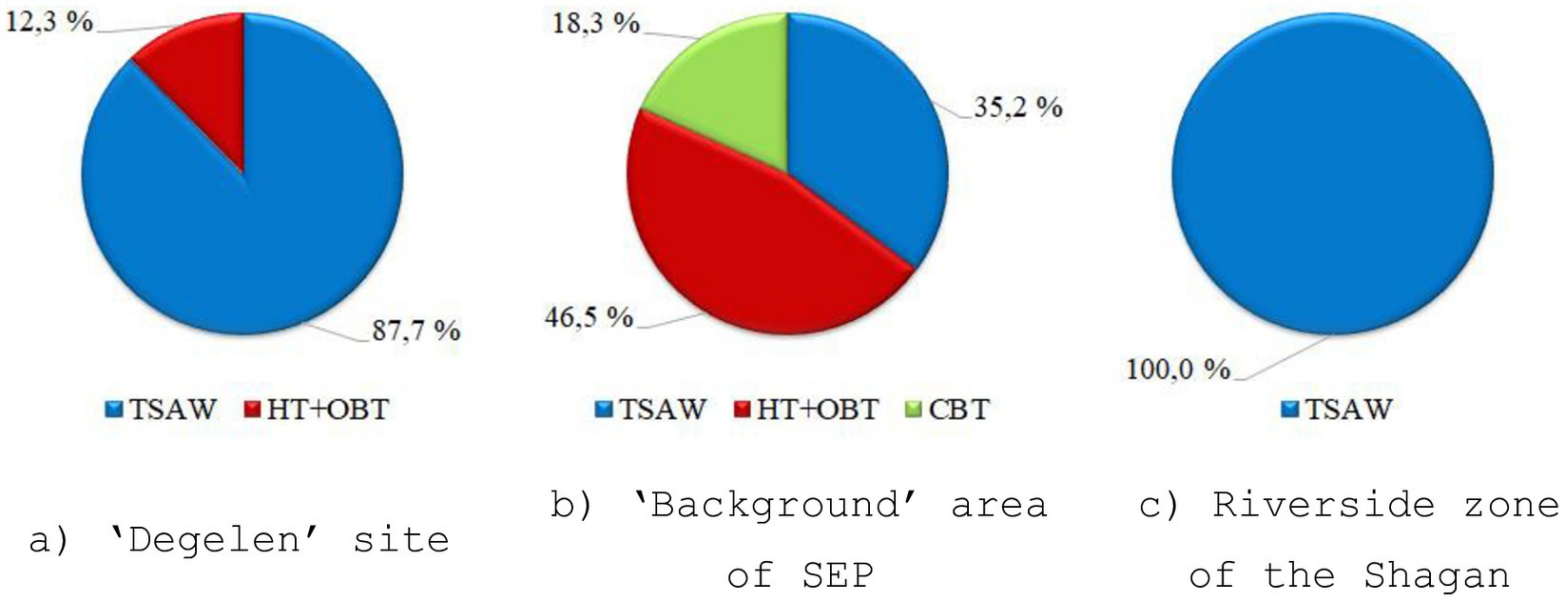
Average content of ^3^H species in the soil, %. a) ‘Degelen’ site, b) ‘Background’ area of SEP, c) Riverside zone of the Shagan.

Water was sampled (Fig 4 in S1 Appendix) monthly (June, July, August) by one sample from creek surfaces at the ‘Degelen’ site and from the Shagan bed. Water in SEP was sampled from surface lakes once (in July). These lakes are recharged by precipitation. In a dry season, lakes dry up, producing dry closed hollows that are covered in white crusts of saline soils and salts. In this regard, no water sampling seemed possible every month.

For transportation and storage, each water sample was collected in plastic canisters.

Soil over the entire research period was sampled once in July. To do so, test pits were drilled at research spots as deep as possible, and depending on the area selected, the depth varied from approximately 100 to 400 cm (Fig 5 in S1 Appendix). Deeper drilling was impossible due to the outcropping bedding rock.

For transportation and storage, each collected soil sample was packed in a triple polyethylene bag. The weight of one sample was about 1 kg.

Air was sampled monthly (June, July, August) for one sample. To that end, a special sampling device was used–a tritium collector ‘OS1700’ (‘ORTEC’, USA), which allows sampling of ^3^H from the air in two forms: in the form of aqueous vapors, HTO, and incorporated in HT gaseous compounds (Fig 6 in S1 Appendix).

The main point of device operation is based upon the sequential capture of ^3^H aqueous vapors with an absorbent placed in special containers: the first cascade captures HTO, and the second cascade, following air oxidation (when passing through a palladium spiral heated up to 450 °C), captures gaseous ^3^H compounds. Distilled water was used as a ^3^H absorbent.

The time of taking one sample was 2 hours. The volume of pumped air for 2 hours was 0.06 m^3^. During air sampling, temperature and humidity were measured using a hygrometer of psychrometric type VIT-2 (JSC ‘Thermopribor’, Russia). During the studies, the air temperature for the whole summer period ranged from 12 to 38.5 °C, humidity—from 10 to 67%.

One of the reasons for conducting studies only in summer time is the limitations of the operational characteristics of the air sampling device used. Since air sampling is possible only at temperatures from + 2 to 45 °C and humidity from 5 to 90%. Also, for such period of time as late fall, winter and early spring there are incomplete conditions for determination of free forms of ^3^H in soil—the soil is too dry.

### 2.3 Laboratory activities

#### 2.3.1 Soil sample preparation

According to the literature data, ^3^H in soil is known to exist in free water and bound forms [17, 32, 33]. Forms such as free water include ^3^H in surface-adsorbed water (TSAW) and ^3^H in interlayer water (TIW) [34]. Organically bound ^3^H (OBT), hydroxyl ^3^H (HT) and crystalline ^3^H (CBT) are bound forms.

Soil samples to determine the content of all ^3^H forms were prepared as per the procedure described in the articles [17, 35] and working instructions [36, 37].

A conventional approach to the determination of radionuclide species is a step-by-step extraction of each form [38].

The articles [17, 35] present methodological approaches for the study of ^3^H forms in soil. The quality and efficiency of the distillation and autoclave decomposition methods used to determine the forms of ^3^H in free water and to isolate bound forms of ^3^H, respectively, are evaluated. The isotope indicator with known specific activity of ^3^H was used during the research. The efficiency of determination of ^3^H content in soil was up to 98%.

^3^H in the surface-adsorbed water was isolated from the sample when heated to 150 °C. ^3^H isolation from the interlayer water requires heating up to 400 °C. Therefore, ^3^H species contained in the form of free water were extracted by distillation [36] with gradually rising temperature. That said, aliquots were collected at 150 °C and 400 °C.

The distillation process (Fig 7 in S1 Appendix) ran in a specially prepared unit consisting of a heating mantle, backflow condenser, and three-necked round-bottomed flask that served as a reactor. A thermometer was mounted in one of the flask necks to control the temperature, and a dry air feed system was mounted in the other. The dry air feed system involved an air feed compressor and a Drexel flask that used calcium chloride as a desiccant. Dried air was fed in order to extract ^3^H completely and accelerate the production of a condensate.

The distillate collected at each temperature was prepared for measurements.

The determination of bound forms requires complete soil digestion, i.e., with the mineral’s crystalline lattice destroyed, which is achievable by autoclave digestion with variations in physical and chemical conditions.

The principle of the autoclave digestion technique consists of decomposing soil samples (with the matrix destroyed) affected by temperature and pressure in the pressurized enclosed volume [37].

The sequential determination of bound forms is impossible. Therefore, the determination process of ^3^H bound forms ran in three stages.

At stage 1 of the autoclave digestion, the total content of ^3^H-bound forms was determined. At stage 2, organically bound ^3^H was removed from the sample by calcination. Calcination was performed in a muffle furnace at 700 °C for 120 min. Next, a calcinated sample was extracted by autoclaving to determine the content of crystalline bound ^3^H. At stage 3, the content of organically bound ^3^H was determined by calculation from the difference in the total content and the content of crystalline bound ^3^H.

*Stage I* Determination of the total content of bound forms

∑=OBT+CBT

*Stage II* Removal of OBT by calcination

$$\sum_{\left(OBT+CBT\right)}\to t_{700\;{}^{\circ}C,\;120\;min}\to CBT$$

*Stage III* Determination of OBT content from the difference in the total content of bound forms and CBT

$$OBT=\sum_{\left(OBT+CBT\right)}-CBT$$

Autoclave digestion was accomplished as follows (Fig 8 in S1 Appendix). A 2 g subsample that was quantitatively transferred to a Teflon reaction chamber (the inner autoclave insert) was collected from a dry sieved soil sample (1 mm). It was treated in concentrated nitric and hydrofluoric acids at a ratio of 3 and 7 ml. The Teflon reaction chamber was placed in the outer autoclave case. Autoclaves were placed in a steel clamping mechanism (bed) to keep them still. Autoclaves were aged in the order of 2.5 hours ion the baker at 160 ± 5 °C. Once autoclaving was complete, a resulting sample was removed from the baker, aged to cool to room temperature and filtered. Insert walls during filtration were flushed in concentrated nitric and hydrofluoric acids to ensure the best wipe sampling.

After autoclave decomposition the obtained sample was a mixture of acids, which made it difficult to measure by liquid scintillation method. For this reason, this called for sample neutralization followed by distillation. To do so, a resulting sample was neutralized with a 50% sodium hydroxide solution to pH = 7–8 and was further distilled. To remove highly volatile products, the first distillate portion (one-fifth of a sample) was discarded. The remaining solution was distilled until dry salts were produced. A resulting condensate was prepared in full for the measurement.

Hydroxyl ^3^H was not isolated. This form was expected to be removed together with organically bound ^3^H, i.e., in calcination.

#### 2.3.2 Water and air sample preparation

Because ^3^H sampling from the air was accomplished by capturing with a liquid absorbent–distilled water, samples were prepared similarly to water sample preparation [39] (Fig 9 in S1 Appendix). To determine the ^3^H concentration both in the water and the air, all of the samples collected were filtered through an ashless filter (for quantitative and qualitative analyses, TC 2642-001-68085491-2011) to remove coarse suspensions.

Once filtered, each sample was placed in a round-bottomed flask and mounted on the heating mantle to remove salts by distillation. A resulting distillate was used as an aliquot for the analysis.

#### 2.3.3 Spectrometric analysis

^3^H in all samples (soil, water and air) was determined as per the procedure [39]. A 5 cm^3^ aliquot was collected from each sample prepared and placed in a 20 cm^3^ plastic vial. A 15 cm^3^ ‘Ultima Gold’ (‘PerkinElmer’, USA) scintillator was added to each vial. The vial was then plugged, and the resulting solution was thoroughly shaken until it became homogeneous.

Samples prepared were measured by liquid scintillation method using ‘TRI-CARB 2900 TR’ (‘PerkinElmer’, USA) spectrometric equipment (Fig 10 in S1 Appendix). A set of ^3^H calibration sources—quenched standards (Packard, Canberra Company, USA) with known ^3^H activity was used for equipment calibration.

The time from water and air sampling to measurement of ^3^H concentrations was on the order of 3 days, all soil samples were on the order of 10 days.

#### 2.3.4 Processing of results

The specific activity of ^3^H in soil was calculated using the following formula:

$$\text{A}\;in\;soil=\left(\left(\frac{CPM-CPMscint}{Vsample\cdot 60\cdot Eff}\right)\cdot 1\;000\right)\cdot \frac{Vdist}{\text{Msubsample}},\;\text{Bq}/\text{kg}$$

where:

CPM—number of pulses per minute registered by the spectrometric equipment ‘TRI-CARB 2900 TR’ for the measured sample, imp/min;

CPMscint—number of pulses per minute, registered by spectrometric equipment ‘TRI-CARB 2900 TR’ for ‘Ultima Gold’ scintillator, imp/min;

1 000—conversion of the value from ml (mg) to l (kg);

Vsample—volume of the measured sample (5 ml);

60—conversion from minutes to seconds (from imp/min to imp/s), since 1 Bq is one decay per second;

Eff– ^3^H registration efficiency, determined by the formula:

$$Eff=\frac{CPM-CPM\;scint}{Aknown\cdot 60}$$

where:

Aknown—known activity of the calibration ^3^H source, Bq;

Vdist—volume of soil distillate;

Msubsample—mass of soil suspension (for bound forms 2 g, for free forms 200 g).

The specific activity of ^3^H in water was determined by the standard formula:

$$\text{A}\;in\;water=\frac{CPM-CPM\;scint}{Vsample\cdot 60\cdot Eff}\cdot 1\;000,\;\text{Bq}/\text{kg}$$

Vsample—volume of measured water sample (5 ml);

The minimum detectable activity (MDA) for the determination of ^3^H in soil and water was calculated by the formula:

$$MDA=2\cdot \frac{\surd \left(CPMfon\cdot t\right)}{t\cdot 60\cdot Eff\cdot Vsample}\cdot 1\;000,\;\text{Bq}/\text{kg}$$

where:

CPMfon—number of pulses per minute registered by spectrometric equipment ‘TRI-CARB 2900 TR’ for pure distilled water, imp/min;

t—measurement time, min.

Volumetric activity of ^3^H in air was calculated by the following formula:

$$\text{A}\;in\;air=\frac{CPM-CPMscint}{Vair\cdot 60\cdot Eff},\;\text{Bq}/{\text{m}}^{3}$$

where:

Vair—volume of pumped air (0.06 m^3^).

MDA for the determination of ^3^H in air was calculated by the formula:

$$\text{M}DA=2\cdot \frac{\surd \left(CPMfon\cdot t\right)}{t\cdot 60\cdot Eff\cdot V_{\text{BO}3д}},\;\text{Bq}/{\text{m}}^{3}$$

The uncertainty was calculated taking into account the standard deviation of the dialed pulses in the spectrum of the measured sample and taking into account the errors of the laboratory measuring utensils used at all stages of sample preparation. On average, the uncertainty amounted to 20%.

^3^H is able to migrate in environmental compartments both horizontally and vertically and downward and backward. After HTO enters the atmosphere, it becomes mixed with air humidity, followed by transfer to or deposition onto the soil. HTO settles in the soil as a result of airborne wet and dry depositions. Thus, the HTO concentration that settled in the soil moisture as per [40–42] is calculated by the following expression:

Csw=CRs−a⋅Cam

where *CRs-a*–a reference value (empirical constant);

*Cam* – HTO concentration in the air humidity, Bq/kg.


Cam=Cair/Ha

where *Cair* – HTO concentration in the air, Bq/m^3^;

*Ha*–the absolute humidity, kg/m^3^ (calculated given the air temperature, °C and air humidity, %; shows the maximum amount of water to be contained in 1 m^3^ of air).

Values of *CRs-a* corresponding to IAEA’s recommendations depend on a number of local factors and may vary from 0.23 to 1 [40]. This is determined by the ratio of the HTO concentration in the soil to that in the air humidity.

$$CRs-a=C_{SHTO}/Cam$$

where *C*_*SHTO*_ − HTO concentration in the soil, Bq/kg.

To calculate *CRs-a* values, values of ^3^H activity concentration in the free form (HTO, ^3^H in the surface-adsorbed water) in the soil and values of HTO concentration in the air humidity were used. It is worth noting that all results obtained from research were used for the calculation in simultaneous soil and air sampling in July.

## 3. Results and discussion

### 3.1 The results

A comparative analysis was carried out to quantitatively assess the content of ^3^H in environmental compartments.

Table 1 lists the values of the ^3^H activity concentration in the water for the entire study area.

**Table 1:** ^3^H activity concentrations in the water.

Study area		Activity concentration of ^3^H, Bq/kg		
		June	July	August
		^a^AC±^b^SD	AC±SD	AC±SD
‘Degelen’ site	BMS-1	54 000±5 000	55 000±5 000	57 000±5 000
	BMS-2	54 000±5 000	32 000±3 000	16 500±2 000
	BMS-3	45 000±4 000	42 000±4 000	41 800±4 000
Shagan	BMS-7	1 000±100	1 100±100	1 300±100
	BMS-8	100±10	61 500±6 000	20 000±2 000
	BMS-9	150±10	8 500±800	10 250±1 000
SEP	BMS-4	-	^c^below MDA	-
	BMS-5	-	below MDA	-
	BMS-6	-	below MDA	-

^a^AC: activity concentrations of ^3^H

^b^SD: standard deviation up to 20%

^c^below MDA: minimum detectable activity

As shown in the table, throughout the summertime, the ^3^H activity concentration in the water of the study area at the ‘Degelen’ site varies from 16 500 to 57 000 Bq/kg. At the BMS-2 site, a decrease in the ^3^H concentration is noted from June through August at 54 000 to 16 500 Bq/kg. Most likely, this is related to the seasonal pattern of the surface water stream in Baitless Creek [43]. For this reason, variations in the water level occur throughout the summertime in the riverside zone of the Shagan river. The Shagan river has ^3^H activity concentrations varying from 100 to 61 500 Bq/kg. At the same time, consistently elevated ^3^H values in the water are characteristic of ‘Degelen’ water streams. The vicinity of the Shagan exhibits the interval of locally growing and dropping ^3^H activity concentrations (BMS-8), which may indicate that surface waters may most likely become contaminated due to the entry of ground waters in this research area [29]. At spots of the conventionally ‘background’ area in SEP of STS, ^3^H in the water was not discovered, and its activity concentration value was below MDA (<6 Bq/kg).

Data on HTO and HT volumetric activities in the air are listed in Table 2.

**Table 2:** HTO and HT volumetric activities in the air.

Study area		^3^H volumetric activity in the air, Bq/m^3^					
		June		July		August	
		HTO	HT	HTO	HT	HTO	HT
		AC±SD	AC±SD	AC±SD	AC±SD	AC±SD	AC±SD
‘Degelen’ site	BMS-1	29±6	11±2	41±8	0.8±0.1	4.6±0.9	0.8±0.1
	BMS-2	11±2	8±1	24±6	0.8±0.1	1.3±0.3	below MDA
	BMS-3	56±11	16±3	46±9	2.3±0.5	16±3	0.9±0.2
Shagan	BMS-7	below MDA	below MDA	below MDA	below MDA	4.1±0.5	below MDA
	BMS-8	4.1±0.5	1.8±0.5	5.5±0.5	below MDA	5.7±0.5	below MDA
	BMS-9	4.9±1.0	6.4±1.3	3.4±0.7	below MDA	0.8±0.1	below MDA
SEP	BMS-4	3.9±0.8	2.5±0.5	below MDA	below MDA	below MDA	below MDA
	BMS-5	4.5±0.9	4.2±0.8	below MDA	below MDA	below MDA	below MDA
	BMS-6	3.8±0.8	1.3±0.3	below MDA	below MDA	below MDA	below MDA

According to the findings, each summer month exhibits an elevated concentration of HTO in the air of the ‘Degelen’ site compared to other research sites. The volumetric activity of HTO was up to 56 Bq/m^3^, and that of HT was up to 16 Bq/m^3^. The maximum HTO volumetric activity in the air of the ‘Degelen’ site is noted in the vicinity of Karabulak (BMS-1) and Uzynbulak cr. (BMS-3) beds, minima–in the vicinity of the Baitles cr. (BMS-2) bed. The maximum HT concentration in the air was registered virtually at every spot only in June.

Values of HTO volumetric activities in the riverside zone of the Shagan are traceable virtually at all research spots throughout the summertime up to 5.7 Bq/m^3^. HT volumetric activity for this area was only revealed in June up to 6.4 Bq/m^3^. The MDA value for determining the volumetric activity of HTO and HT in air is 0.5 Bq/m^3^.

Within SEP, numerical values of HTO and HT were only recorded in June (up to 4.5 Bq/m^3^).

Activity concentration values obtained for ^3^H species in the soil are listed in Table 3.

**Table 3:** Activity concentrations of ^3^H species in the soil.

Study area		Soil sampling depth, cm	^3^H activity concentration, Bq/kg			
			^3^H in surface-adsorbed water (TSA)	^3^H in interlayer water (TIW)	Hydroxyl+or-ganically bound ^3^H (HT+OBT)	Crystal-line bound ^3^H (CBT)
			AC±SD	AC±SD	AC±SD	AC±SD
‘Degelen’ site	BMS-1	0–40	5 170±500	below MDA	340±30	below MDA
	BMS-2	0–100	120±12	below MDA	230±20	below MDA
		100–200	1 820±180	below MDA	270±30	below MDA
		200–300	560±50	below MDA	190±20	below MDA
		300–360	840±80	below MDA	190±20	below MDA
	BMS-3	0–100	1 840±180	below MDA	below MDA	below MDA
Shagan	BMS-7	0–80	55±6	below MDA	below MDA	below MDA
		80–160	170±17	below MDA	below MDA	below MDA
Shagan	BMS-8	0–120	4 100±400	below MDA	below MDA	below MDA
Shagan	BMS-9	0–100	100±10	below MDA	below MDA	below MDA
		100–200	60±6	below MDA	below MDA	below MDA
		200–280	55±6	below MDA	below MDA	below MDA
SEP	BMS-4	0–100	4±1	below MDA	below MDA	below MDA
		100–200	6±1	below MDA	below MDA	below MDA
		200–300	30±12	below MDA	220±20	below MDA
SEP	BMS-5	0–100	12±1	below MDA	below MDA	below MDA
		100–200	11±2	below MDA	1 710±170	below MDA
		200–350	8±1	below MDA	1 470±150	below MDA
SEP	BMS-6	0–150	22±2	below MDA	1 000±100	1 250±130

In the course of the analysis, ^3^H in the soil of every research site was found to be mostly in the form of surface-adsorbed water, which is one of the free forms. No other free form– ^3^H in the interlayer water–was observed at any of the research spots. Maxima of ^3^H activity concentrations in the form of surface-adsorbed water are noted for the soil of the ‘Degelen’ site and the riverside zone of the Shagan riv. The concentration of this form varied from 120 to 5 170 Bq/kg and from 55 to 4 100 Bq/kg, respectively. That said, the distribution in the soil profile is nonuniform. For example, at the ‘Degelen’ site (BMS-2), the maximum value is noted in the 100–200 cm layer and in the vicinity of Shagan in the 80–160 cm (BMS-7) and 0–120 cm (BMS-8) layers. The ‘background’ area of SEP is notable for the lower level of ^3^H activity concentration– 4 to 30 Bq/kg.

^3^H-bound forms were revealed in the soil of the ‘Degelen’ site and in the ‘background’ area of SEP. Values of ^3^H activity concentrations in the hydroxyl and organically bound forms for the ‘Degelen’ site were up to 340 Bq/kg, and those for the ‘background’ area of SEP were up to 1 700 Bq/kg. For the riverside zone of the Shagan, ^3^H in this form was below MDA—<50 Bq/kg. ^3^H in the crystalline bound form was detected only in one case in the ‘background’ area of SEP at BMS- 6 with an activity concentration value of 1.250 Bq/kg.

### 3.2 Discussion

The figure (Fig 2) graphically presents the dynamics of the average ^3^H content in the water by months of summertime observations.

As seen from the graphs presented, as a whole, no seasonal variation in ^3^H activity concentration is observed on the boundary of the ‘Degelen’ site. Some decrease is only characteristic of BMS-2 (Table 1). Unlike the ‘Degelen’ site, an increase in the ^3^H concentration from June to August is characteristic of Shagan water. The exception is BMS-7, where the ^3^H concentration over the observation period is noted to be 1 000–1 300 Bq/kg, which is within the determination uncertainty of ^3^H (Table 1). As opposed to ‘Degelen’, in general, a much wider range of ^3^H concentration values is observed during the observation period. At the BMS-8 spot, an interesting dynamic of ^3^H content (Table 1) can be traced. The ^3^H activity concentration from a minimum in June of 100 Bq/kg increased to a maximum of 61 500 Bq/kg in July. In August, the ^3^H concentration decreased to 20 000 Bq/kg (Table 1). Such dynamics of ^3^H concentration at this spot may be due to tectonic disturbances revealed earlier by geophysical research [28, 29]. These disturbances are the main channel through which ^3^H-contaminated fracture waters discharge into surface waters of the Shagan river in this particular bed section. It is worth noting that the recharge zone of ground waters is in the northern ‘Balapan’ site approximately 20 km away from point BMP-8. That said ground waters at the ‘Balapan’ site are artesian with a head value of up to 80 meters, for which reason ^3^H-contaminated waters enter the Shagan riv. with a certain head. For example, the higher a head value the more contaminated waters enter the river. At the same time, the maximum total discharge of streams falls behind the onset of intense precipitation event by more than 2 months. That period can be taken as the infiltration time of precipitation through fracture systems at the ‘Balapan’ site from the recharge zone to the discharge area. Early in July, it reaches maxima and by the end of the month, it begins to decrease. In this regard, in June we measure low values of ^3^H concentration (100 Bq/kg) and a sharp increase in ^3^H activity concentration up to 61 500 Bq/kg in July. In August ^3^H concentration is recorded to decrease down to 20 000 Bq/kg due to a lower ground water head (Table 1).

Figures (Fig 3) below depict the dynamics of the average concentrations of HTO and HT in the air of research sites versus sampling months.

As mentioned above, air was sampled along water streams–creeks of the ‘Degelen’ site and the Shagan river Air is closely linked to open tritium sources, specifically, soil and water, in which the process of nonuniformly emanated ^3^H is sure to occur from surfaces of sources throughout the year depending on the time period. Vegetation growing at research spots can be an additional open source of ^3^H –the transpiration process may also affect the mechanism by which ^3^H is produced in the air [6].

Maxima of HTO and HT volumetric activity in the air were registered at the ‘Degelen’ site, 56 and 16 Bk/m^3^, respectively. It is worth noting that at this site, maxima of ^3^H activity concentration were also registered in the water and soil. The dynamics of the HTO concentration in the air show that the values of the volumetric activity decrease toward August, which in specific cases is characteristic of surface waters–BMS-2 (Table 1, Fig 2). Such a variation in HTO in the air may be affected by a decrease in the temperature at this point in time followed by a reduced evaporation of ^3^H from water, its emanation from soil and transpiration from vegetation.

If the dynamics of the HTO concentration in the air are considered for the riverside zone of the Shagan, one can trace an increasing trend of the HTO concentration from June to August, which is consistent with an increase in the ^3^H concentration in the water (Fig 2). The exception is BMS-9, which in this case may be attributed to the location of this site on the right bank with the inflow of purer ground waters [44]. That said, the nonuniform entry of HTO into the air of BMS-7 and BMS-8 may be impacted by the capillary rise of ^3^H-contaminated ground waters coming from the ‘Balapan’ site followed by evaporation from the soil surface.

The study of HTO concentration in the ‘background’ area of SEP and HT concentration at all other research spots revealed the same picture of their dynamics. All of the numerical values of ^3^H volumetric activity were registered in June, followed by a sharp decrease in the concentration down to the detection limit of 0.5 Bq/m^3^. At the same time, no quantitative values of ^3^H in the water of this territory were revealed (Table 1). Such a change in HTO for SEP is attributable to one of the reasons. This area is mainly characterized by saline soils. July is believed to be the hottest month when a drought season occurs. By that time, all of the sites available, such as lakes, plants and soil, dry up in this area for which reason ^3^H emanation from the soil and its transpiration from plants cease. Hence, in SEP, numerical values of HTO volumetric activity in the air are only registered in July.

The dynamics of HT concentration in the air of all research spots demonstrates a unified behavior of distribution–maxima of the volumetric activity at all research spots were noted in July. At the same time, these are only quantitatively recorded throughout the research period on the boundary of the ‘Degelen’ site. Perhaps this is due to intrazonal conditions of the mountain range–the presence of ample meadow vegetation, which produces up to 500 g/m^2^ [7] and soils with high humus content–up to 20% [27]. That said, the entry and production of HT in the air in the first summer month may be affected by vital activities of microorganisms in the soil and the plant-animal organic mass containing ^3^H [9]. The ratio between heat and moisture is known to be a determinant of the maximum number and vital activities of microorganisms in the soil [45]. Most likely, late spring and early summer account for such an optimal ratio. In that period, the temperature is not high with rainfall, which is necessary for maintaining vital activities of microorganisms in the soil and the plant-animal organic mass. The appearance and production of the maximum HT concentration in June is most likely to fall just on when complex organic matter contained in the soil is split into more elementary matter capable of being emanated, producing volatile gaseous compounds, which, among others, contain gaseous ^3^H compounds [9]. As soon as the hot spell comes, the number and vital activities of microorganisms in the soil are reduced and slowed down. For this reason, the HT concentration in the air decreases by midsummer.

Because soil over the entire research period was only sampled once, any graph constructions to trace the dynamics of ^3^H content in the soil seem impossible. For a more detailed concept of a ^3^H form in the soil, its species have been studied. The diagrams (Fig 4) depict the average percentage of ^3^H species detected in the soil of all research spots.

Diagrams derived show the percentage of ^3^H species in the soil. The soil of the ‘Degelen’ site is dominated by free water forms, namely, by ^3^H in the surface-adsorbed water. The average percentage of ^3^H in the surface-adsorbed water all over the ‘Degelen’ site was on the order of 88%. The total content of bound forms is insignificant and does not exceed 12%.

The soil of the SEP ‘background’ area is dominated by ^3^H bound forms, in which the average percentage is on the order of 65% for the entire ‘background’ area. Of these, at one spot (BMS–6), the content of crystalline bound ^3^H is observed in the soil, whereas other spots (BMS-4 and BMS-5) contain no form like this but are dominated by organically bound ^3^H. The average content of ^3^H in the surface-adsorbed water was 35%.

Determinations of ^3^H species in the soil of the Shagan riverside zone showed that ^3^H at all BMSs is only contained in the surface-adsorbed water.

Based upon the results presented, it is worth noting that free water ^3^H dominates in the soil of the research spots. This applies specifically to the ‘Degelen’ site and the Shagan riverside zone with research spots located in the bed of 3 water streams (Karabulak, Baitless and Uzynbulak cr.) and along the Shagan bed, respectively. ^3^H domination in the form of free water is most likely to be related to the inflow of ground waters in the soil. ^3^H domination, according to hydrogeological maps of the terrain, occur at shallow depths on the order of 2–5 m along water streams. The presence of rock fragments along the bed from underground nuclear explosions in mountain adits may affect ^3^H bound forms fixed in the soil of research spots at the ‘Degelen’ site [27]. Rock is a mineral in which hydroxyl ^3^H (chemically bound water) can be incorporated [17]. The presence of ^3^H in bound forms is more difficult to explain in the ‘background’ area, which is at a distance from nuclear test locations. The radiological situation in this territory is attributed to the fallout from aboveground nuclear tests conducted at the ‘Experimental Field’ and ‘Telkem’ sites. While the ^3^H concentration in the soil in the form of free water is available in the source, the concentration in the organic constituent may reflect ^3^H historical levels in the environment years or decades ago [46, 47]. It is worth noting that in the course of earlier research undertaken in the SEP ‘background’ area, the presence of ^3^H could be recorded in organic constituents of plants [31].

Table 4 lists the *CRs-a* values and HTO concentrations in the soil moisture (*Csw*) for all BMSs of interest.

**Table 4:** CRs-a and Csw values for all BMS of interest.

Name of a research spot		TSAW, Bq/kg	CRs-a	Csw, Bq/kg
‘Degelen’ site	BMS-1 (*n = 1)	5170.0	1.1	5173.8
	BMS-2 (n = 4)	835.8	0.2	857.1
	BMS-3 (n = 1)	1835.0	0.4	1840.0
Shagan riverside zone	BMS-7 (n = 2)	114.0	1.9	114.3
	BMS-8 (n = 1)	4133.0	7.1	4133.7
	BMS-9 (n = 3)	71.7	0.3	72.9
SEP	BMS-4 (n = 3)	13.3	0.3	13.2
	BMS-5 (n = 3)	10.3	0.1	10.0
	BMS-6 (n = 1)	22.0	0.3	21.9

*n–number of soil layers sampled during pits drilling

The table lists reported HTO concentrations in the soil moisture Csw calculated given *CR s- a* values and HTO concentrations in the air humidity *Cam*. From the results, one can see a quite low but additional contribution by HTO to the soil from the air.

*CRs-a* ratios that were calculated became certain values for each specific research spot. The value interval is 0.1 to 7.1. The spread of values can be attributed to the variable pattern of the source of ^3^H entry into different environmental compartments over the entire summertime of research efforts. It was noted that many reported values of *CRs-a* are within the value limit as per IAEA’s recommendations (from 0.23 to 1). Exceptions are the values of 1.9 and 7.1 calculated for the BMS-7 and BMS-8 spots. Because the ratio of the HTO concentration in the soil to that in the air humidity *CRs-a* may depend on a number of factors [40], the resulting values are most likely associated with the presence of a developed zone of groundwater containing ^3^H in the coastal zone of the Shagan riv. (the area of the 2nd and 5th km from the ‘Atomic Lake’, respectively). The mechanism of ^3^H distribution in such a system can be caused by the processes of capillary rise of polluted groundwater followed by evaporation from the soil surface. The maximum value of 7.1 may additionally be because in July, the specific activity of ^3^H in water at the BMS-8 study site was 61 500 Bq/kg (Table 1). This is the highest ^3^H value for water over this research period, which is related to the discharge of ^3^H-contaminated fracture waters into surface waters of the Shagan river in this bed section as described above. The most stable *CRs-a* values are characteristic of the SEP ‘background’ area, which is apparently related to no direct impact by an open water source. This fact proves that the aquatic medium matters greatly in the migration and distribution of ^3^H in various environmental compartments.

## 4. Conclusion

Activities carried out at STS showed the presence of ^3^H in different forms in environmental compartments of interest. ^3^H was recorded not only in locations of underground nuclear tests but also in the ‘background’ area–STS’s SEP. Maximum ^3^H tritium concentrations in the water were detected at the ‘Degelen’ site (up to 57 000±5 000 Bq/kg) and the Shagan riv (up to 61 500±6 000 Bq/kg), in the air of the ‘Degelen’ site (up to 56±11 Bq/m^3^), in the soil of the ‘Degelen’ site (up to 5 170±500 Bq/kg) and the Shagan riv (4 100±400 Bq/kg) in the free water, at SEP (up to 1 710±170 Bq/kg) in the organic constituent. Research undertaken suggests that water plays a key role in migration processes of ^3^H in the natural environment of interest. The second most but equally important constituent is soil and all microorganisms of plant and animal origin living there. These assumptions are indirectly proven by research findings showing the dynamics of HTO and HT concentrations in the air versus a sampling month. In the soil of research spots, the bulk of ^3^H is contained in the form of free water. This form of ^3^H is predominant where ^3^H migrates with surface waters (creeks of the ‘Degelen’ site and the Shagan riv.). ^3^H bound forms were also revealed in the soil of the ‘Degelen’ site and in the ‘background’ area. Accordingly, rock fragments following underground nuclear explosions conducted at the ‘Degelen’ site can be mechanisms by which these forms are produced, and the fallout from aboveground nuclear tests conducted at the ‘Experimental Field’ and ‘Telkem’ sites can be the ‘background’ area.

As all efforts progressed, ^3^H in the STS area was found to be readily distributed in abiotic environmental compartments depending on several factors. Not only the radiation effect after nuclear testing but also the following can be the major contributors to ^3^H distribution: weather conditions (air temperature/humidity), seasonal changes, the type and state of the land cover, hydrogeological changes leading to the variation in ^3^H behavior in surface waters, transpiration from plants, vital activities of microorganisms in the soil and others [1].

Ratios of the HTO concentration in the soil to that in the air humidity *(CRs-a)* were calculated. These, in most cases, correspond to IAEA values. The transfer of ^3^H to the soil from the air in the form of HTO through airborne wet and dry depositions was determined as an additional contribution.

Findings must be considered in monitoring areas, for instance, near NPPs and other radiation-hazardous objects, to assess the impact of ^3^H releases on the public and environment to avoid underestimation.

## Supporting information

S1 Appendix (DOC)

## References

[1] Antonova EV, Antonov KL, Vasyanovich ME, Panchenko SV, 2022. Tritium from the molecule to the biosphere. Russian Journal of Ecology. Vol. 53. 253–284. doi: 10.1134/S1067413622040038

[2] Baturin VA., 2001. Tritium is dangerous. Support Center of Civil Initiatives. Chelyabinsk. 58.

[3] Ozharovsky A. Tritium around NPP, 2013. https://bellona.ru/2013/12/12/na-kalininskoj-aes-tritij-zakachivayut/

[4] Aidarkhanov AO, Lukashenko SN, Subbotin SB, Edomin VI, Genova SV, Toporova AV, et al, 2010. Shagan and the major mechanism by which it is produced. Topical issues in radioecology of Kazakhstan. Proceedings of the Institute of Radiation Safety and Ecology over 2007–2009. Issue 2. Pavlodar. 9–54. ISBN 978-601-7112-32-5.

[5] Aktayev MR, Lukashenko SN, Aidarkhanov AO, Lyahova ON, 2017. The contamination pattern of the Shagan water with tritium in the vicinity of the ‘Atomic Lake’. Topical issues in radioecology of Kazakhstan. Proceedings of the National Nuclear Center RK. Issue 6, Vol. I. Pavlodar. 202–207. ISBN 978-601-7844-53-0.

[6] Lyakhova ON, Lukashenko SN, Larionova NV, Tur YS, 2012. Contamination mechanisms of air basin with tritium in venues of underground nuclear explosions at the former Semipalatinsk test site. J. Environ. Radioact. Vol.113. 98–107. doi: 10.1016/j.jenvrad.2012.02.010 22672895

[7] Lyakhova ON, Lukashenko SN, Mulgin SI, Zhdanov SV, 2013. Tritium as an indicator of venues for nuclear tests. J. Environ. Radioact. Vol. 124. 13–21. doi: 10.1016/j.jenvrad.2013.03.004 23639690

[8] Lyakhova ON, Lukashenko SN, Timonova LV, Burdakina OV, 2017. Current activity levels of artificial radionuclides in objects of water use located at the territory of the Semipalatinsk Test Site (STS). Radiation biology. Radioecology. Vol. 57. No. 1. 77–85. doi: 10.7868/S0869803117010106 30698935

[9] Lyakhova ON, Lukashenko SN, Timonova LV, Larionova NV, Turchenko DV, 2020. Assessment of the concentration level of gaseous tritium compounds at nuclear test locations of the Semipalatinsk Test Site. Radiation biology. Radioecology. Vol. 60. No. 6. 646–657. doi: 10.31857/S086980312006020X

[10] Marchenko OO, Lyakhova ON, Timonova LV, 2020. Determination of 3H contribution to radiation exposure of personnel and the public when it enters the Semipalatinsk Test Site with air. Bulletin of NNC RK. No. 4 (84). 18–23. ISSN 1729-7516.

[11] Larionova NV, Lukashenko SN, Lyakhova ON. et al., 2017. Plants as indicators of tritium concentration in ground water at the Semipalatinsk test site. J. Environ. Radioact. Vol. 177. 218–224. doi: 10.1016/j.jenvrad.2017.06.032 28711773

[12] Polivkina YeN, Larionova NV, Lyahova ON, 2020. Assessment of tritium uptake by Helianthus Annuus culture continuously exposed to HTO at the Semipalatinsk test site. Radiation and Risk. Vol. 29, No. 1. 79–89. doi: 10.21870/0131-3878-2020-29-1-79-89

[13] Polivkina YeN, Larionova NV, Lukashenko SN, Lyakhova ON, Abisheva MT, Subbotina LF, et al, 2021. Assessment of the tritium distribution in the vegetation cover in the areas of underground nuclear explosions at the Semipalatinsk test site. J. Environ. Radioact. Vol. 237, 106705. doi: 10.1016/j.jenvrad.2021.106705 34329852

[14] Timonova LV, Lyakhova ON, Lukashenko SN, Aidarkhanov AO, 2015. Research into the content of tritium in the soil at nuclear test locations of the Semipalatinsk Test Site. Radiation biology. Radioecology. Vol. 55, No. 6. 667–672. doi: 10.7868/S0869803115050136 26964352

[15] Serzhanova ZB, Aidarkhanova AK, Lukashenko SN, Lyakhova ON, Timonova LV, Raimkanova AM, 2018. Researching of tritium speciation in soils of ‘Balapan’ site. J. Environ. Radioact. Vol. 192. 621–627. doi: 10.1016/j.jenvrad.2018.02.016 29519578

[16] Timonova LV, Lyakhova ON, Lukashenko SN, Aidarkhanov AO, Kabdyrakova AM, Serzhanova ZB, 2020. Tritium distribution in soil in the area of the ‘Atomic Lake’ near the Semipalatinsk Test Site. Eurasian Soil Science. Vol. 53, No. 3. 355–361. doi: 10.1134/S1064229320030096

[17] Serzhanova ZB, Aidarkhanova AK, Lyakhova ON, Timonova LV, Raimkanova AM, 2020. Methodological approaches to the study of tritium bound species in soils of radioactively contaminated areas at the Semipalatinsk Test Site. Bulletin of NNC RK. No. 2. 41–48. ISSN 1729-7516.

[18] Timonova LV, Lyakhova ON, Aidarkhanov AO, Serzhanova ZB, 2020. Tritium-contaminated soil at aboveground nuclear test locations of the Semipalatinsk Test Site. Radiation and risk. Vol. 29. No. 4. 106–117. doi: 10.21870/0131-3878-2020-29-4-106-117

[19] Turchenko DV, Lukashenko SN, Aidarkhanov AO, Lyakhova ON, 2014. Studying of tritium content in the snowpack of the Degelen mountain range. J. Environ. Radioact. Vol. 132. 115–120. doi: 10.1016/j.jenvrad.2014.01.017 24657814

[20] Kulsartov T., Zaurbekova Z., Tazhibayeva I., Ponkratov Y., Gnyrya V., Kizane G., et al. Study of tritium and helium generation and release from lead-lithium eutectics Li15.7Pb under neutron irradiation. – Fusion Engineering and Design. – Vol. 146. – 2019. – P. 1317–1320. doi: 10.1016/j.fusengdes.2019.02.066

[21] Kulsartov T., Shaimerdenov A., Zaurbekova Z., Kenzhina I., Chikhray Y., Kizane G., et al. Features of the in-situ experiments on studying of tritium release from lithium ceramic Li2TiO3 using vacuum extraction method. – Fusion Engineering and Design. – Vol. 172. – 2021. – №. 112703. doi: 10.1016/j.fusengdes.2021.112703

[22] Kulsartov T., Kenzhina I., Chikhray Y., Zaurbekova Z., Kenzhin Y., Aitkulov M., et al. Determination of the activation energy of tritium diffusion in ceramic breeders by reactor power variation. – Fusion Engineering and Design. – Vol. 172. – 2021. – №. 112783. doi: 10.1016/j.fusengdes.2021.112783

[23] Gordienko Y., Ponkratov Y., Kulsartov T., Tazhibayeva I., Zaurbekova Z., Koyanbayev Y., et al. Research Facilities of IAE NNC RK (Kurchatov) for Investigations of Tritium Interaction with Structural Materials of Fusion Reactors. – Fusion Science and Technology. – №6. – Vol. 76. – 2020. – P. 703–709. doi: 10.1080/15361055.2020.1777667

[24] Askerbekov S., Kenzhina I., Kulsartov T., Chikhray Y., Tazhibayeva I., Ponkratov Y., et al. Analysis of reactor experiments to study the transfer processes of generated tritium in lithium cps (capillary-porous system). – International Journal of Hydrogen Energy. – №11. – Vol. 47. – 2022. – P. 7368–7378. doi: 10.1016/j.ijhydene.2021.03.163

[25] Kulsartov T., Kenzhin Y., Knitter R., Kizane G., Chikhray Y., Shaimerdenov A., et al. Investigation of hydrogen and deuterium impact on the release of tritium from two-phase lithium ceramics under reactor irradiation. – Nuclear Materials and Energy. – Vol. 30. – 2022. – №. 101115. doi: 10.1016/j.nme.2022.101115

[26] Lyakhova ON, Lukashenko SN, Subbotin SB, Aidarkhanov AO, Kubenov AM, 2007. Study of the content of tritium in environmental compartments at the ‘Degelen’ testing site. Bulletin of NNC RK. No. 4. 80–86. ISSN 1729-7516.

[27] Panitskiy AV, Lukashenko SN, 2015. Nature of radioactive contamination of components of ecosystems of streamflows from tunnels of Degelen massif. J. Environ. Radioact. Vol. 144. 32–40. doi: 10.1016/j.jenvrad.2015.02.021 25791901

[28] Yesimbekov AZh, Aidarkhanov AO, Aktaev MR, Drozdov AV, 2013. Determination and localization of channels of ^3^H entry into waters of the Shagan river. Topical issues in radioecology of Kazakhstan. Proceedings of the Institute of Radiation Safety and Ecology. Issue 4, Vol. II. 25–40. ISBN 978-601-7112-75-2.

[29] Yesimbekov AZh, Lukashenko SN, Aidarkhanov AO, Aktaev MR, 2017. Research into mechanisms and pathways of surface water contamination with tritium at the Shagan river. Topical issues in radioecology of Kazakhstan. Proceedings of the Institute of Radiation Safety and Ecology. Issue 6, Vol. I. 361–371. ISBN 978-601-7844-53-0.

[30] Lukashenko SN, Strilchuk YuG, Yakovenko YuYu, Turchenko DV, Svetacheva YuV, Umarov MA, et al, 2017. Radioecological state of the ’south-eastern’ part of the STS territory. Topical issues in radioecology of Kazakhstan. Proceedings of the Institute of Radiation Safety and Ecology. Issue 6. Vol. I. 11–90. ISBN 978-601-7844-53-0.

[31] Larionova NV, Lyakhova ON, Serzhanova ZB, Timonova LV, Aidarkhanova AK, Lukashenko SN, 2017. Study of tritium redistribution of in natural ecosystems of the Semipalatinsk Test Site. Nuclear science and technologies: abstracts of the International scientific forum, September 12–15. Almaty. INP NNC RK. 326.

[32] Pushkarev AV, Dolin VV, Priymachenko VV, Bobkov VN, Pushkareva RA, 2007. Kinetics of isotope-hydrogen exchange in bentonite-sand mixture. Institute of Environmental Geochemistry. Kiev. Issue 15. 27–36.

[33] Lopez-Galindo A, Fenoll Hach-Alip P, Pushkarev AV, Lytovchenko AS, Baker JH, Pushkarova RA, 2008. Tritium redistribution between water and clay minerals. Applied Clay Science. № 39. 151–159. doi: 10.1016/j.clay.2007.06.005

[34] Shishelova TI, Sozinova TV, Konovalova AN, 2010. Spectroscopy hands-on training. Water in minerals. ISBN 978-5-91327-093-1. 88.

[35] Serzhanova ZB, Aidarkhanova AK, Lyakhova ON, Koishybaev RA, 2018. Optimization of methods of research of tritium contamination of soils with consideration of the peculiarities of the Semipalatinsk Test Site. Bulletin of NNC RK. No. 4. 49–53. ISSN 1729-7516.

[36] Operating Instruction, 2018. Sample preparation by distillation to determine the content of tritium in free water of soils, grounds and bottom sediments in the Department for the Development of Environmental Monitoring Systems of the branch ‘Institute of Radiation Safety and Ecology’ of RSE NNC RK. No. 01-08/1401. Kurchatov. 13.

[37] Operating Instruction, 2019. Sample preparation by autoclave digestion to determine the content of bound tritium in the soil, ground and bottom sediments in the Department for the Development of Environmental Monitoring Systems of the branch ‘Institute of Radiation Safety and Ecology’ of RSE NNC RK. No. 01-08/639. Kurchatov. 15.

[38] Kabdyrakova AM, Kunduzbayeva AYe, Lukashenko SN, 2010. Species of radionuclides in soils of water stream ecosystems at the Degelen mountain range. Topical issues in radioecology of Kazakhstan. Proceedings of the Institute of Radiation Safety and Ecology. Pavlodar. 285–299. ISBN 978-601-7112-32-5.

[39] ISO 9698:2019 (E). Water quality. Tritium. A liquid scintillation counting technique to determine activities. Third edition 2019–05.

[40] International Atomic Energy Agency (IAEA), 2010. Handbook of Parameter Values for the Prediction of Radionuclide Transfer in Terrestrial and Freshwater Environments. TRS 472. International Atomic Energy Agency, Vienna.

[41] International Atomic Energy Agency (IAEA), 2003. Modelling the Environmental Transport of Tritium in the Vicinity of Long-Term Atmospheric and Sub-surface Sources. IAEA-BIOMASS-3. International Atomic Energy Agency, Vienna.

[42] Davis PA, Kotzer TG, Workman WJG, 2002. Environmental tritium concentrations due to continuous atmospheric sources, Fusion Sci. Technol. 41. 453–457. doi: 10.13182/FST02-A 226630

[43] Subbotin SB, Lukashenko SN, Kashirsky VV, Yakovenko YuYu, Bakhtin LV, 2010. Underground migration of artificial radionuclides beyond the Degelen mountain range. Topical issues in Radioecology of Kazakhstan. Proceedings of the Institute of Radiation Safety and Ecology. Pavlodar. 103–156. ISBN 978-601-7112-32-5.

[44] Subbotin SB, Lukashenko SN, Aidarkhanov AO, Larionova NV, Yakovenko YuYu., 2010. Radioactive contamination of the Shagan ecosystem components with man-made radionuclides. Problems of biogeochemistry and geochemical ecology. No. 3 (14). 113.

[45] Bagirova ChZ, 2021. Basic regularities in the development of bacterial flora in soils of Sumgait, Azerbaidzhan. Science of the South of Russia. Vol. 17, No. 3. 38–46.

[46] Kim SB, Bredlaw M, Korolevych VY, 2013. Organically bound tritium (OBT) in soil at different depths around Chalk River Laboratories (CRL), Canada / AECL Nuclear Rev., 2 (2). 17–26. doi: 10.12943/ANR.2013.00014

[47] Eyrolle-Boyer F, Boyer P, Claval D, Charmasson S, Cossonnet C, 2014. Apparent enrichment of organically bound tritium in rivers explained by the heritage of our past. J. Environ. Radioact., 136., 162–168. doi: 10.1016/j.jenvrad.2014.05.019 24956583

