# Supplementary material for: Tritium distribution in the ‘water-soil-air’ system in the Semipalatinsk Test Site
Source: PLoS One. 2024 Apr 4;19(4):e0297017. doi: 10.1371/journal.pone.0297017 (PMC10994305; doi:10.1371/journal.pone.0297017)
Supplement: S1 Appendix — (DOC) [file pone.0297017.s001.doc]

**Appendix**

| 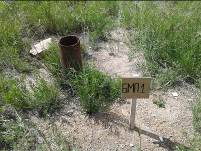 | 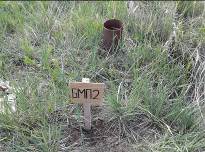 | 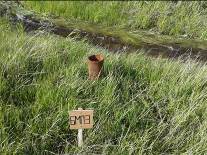 |
| --- | --- | --- |

**Fig 1. Research areas at the ‘Degelen’ site**

| 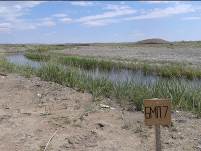 | 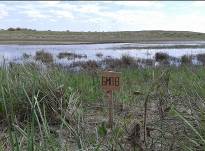 | 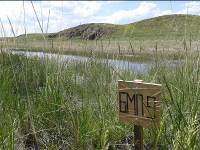 |
| --- | --- | --- |

**Fig 2. Research areas in the riverside zone of the Shagan**

| 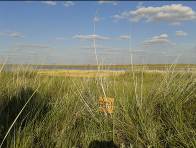 | 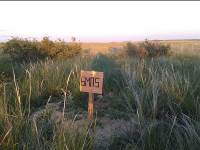 | 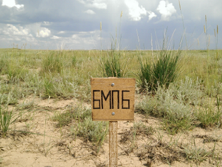 |
| --- | --- | --- |

**Fig 3. Research areas on the conventionally ‘background’ area**

| 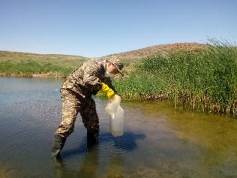 | 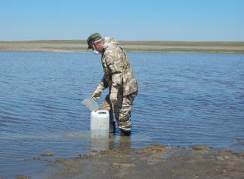 |
| --- | --- |

**Fig 4. Water sampling (Alexey Dashuk)**

| 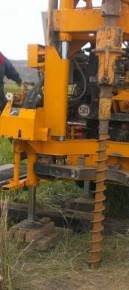 | 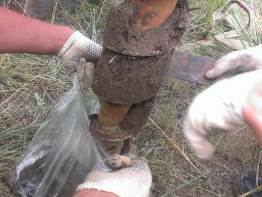 |
| --- | --- |

**Fig 5. Soil sampling**

| 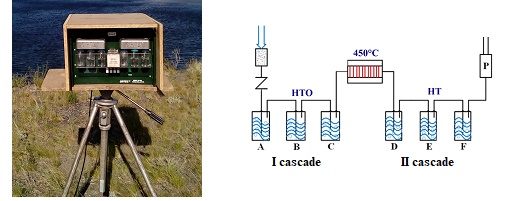 | |
| --- | --- |
| a) General view of the tritium collector “OS1700” | b) Principal scheme of tritium collector operation |

**Fig 6. Sampling device tritium collector ‘OS1700’ for air sampling**


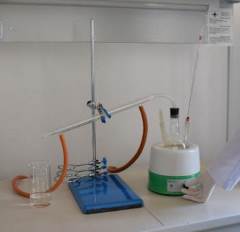


**Fig 7.** **Installation for sample distillation**


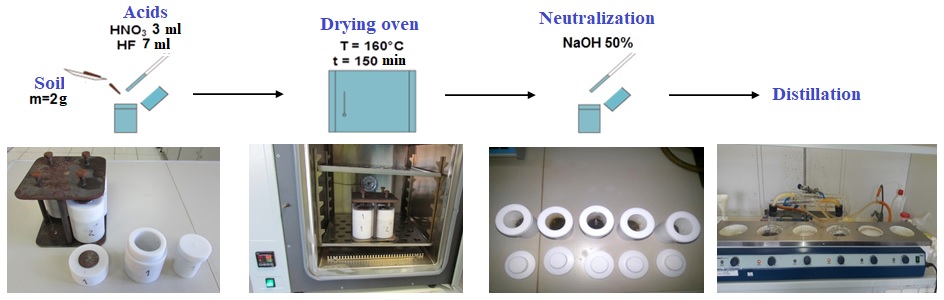


**Fig 8. Scheme of the autoclave soil decomposition process**

| 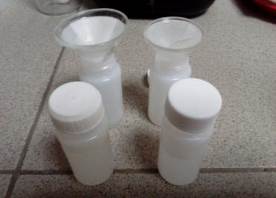 | 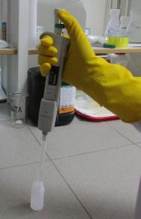 |
| --- | --- |
| a) Filtration of liquid samples | b) Aliquot selection |
| 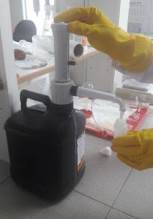 | 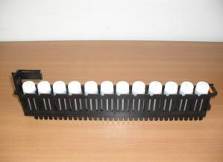 |
| c) Adding a scintillator | d) Prepared samples for measurement |

**Fig 9.** **Stages of sample preparation for measuring tritium concentration**


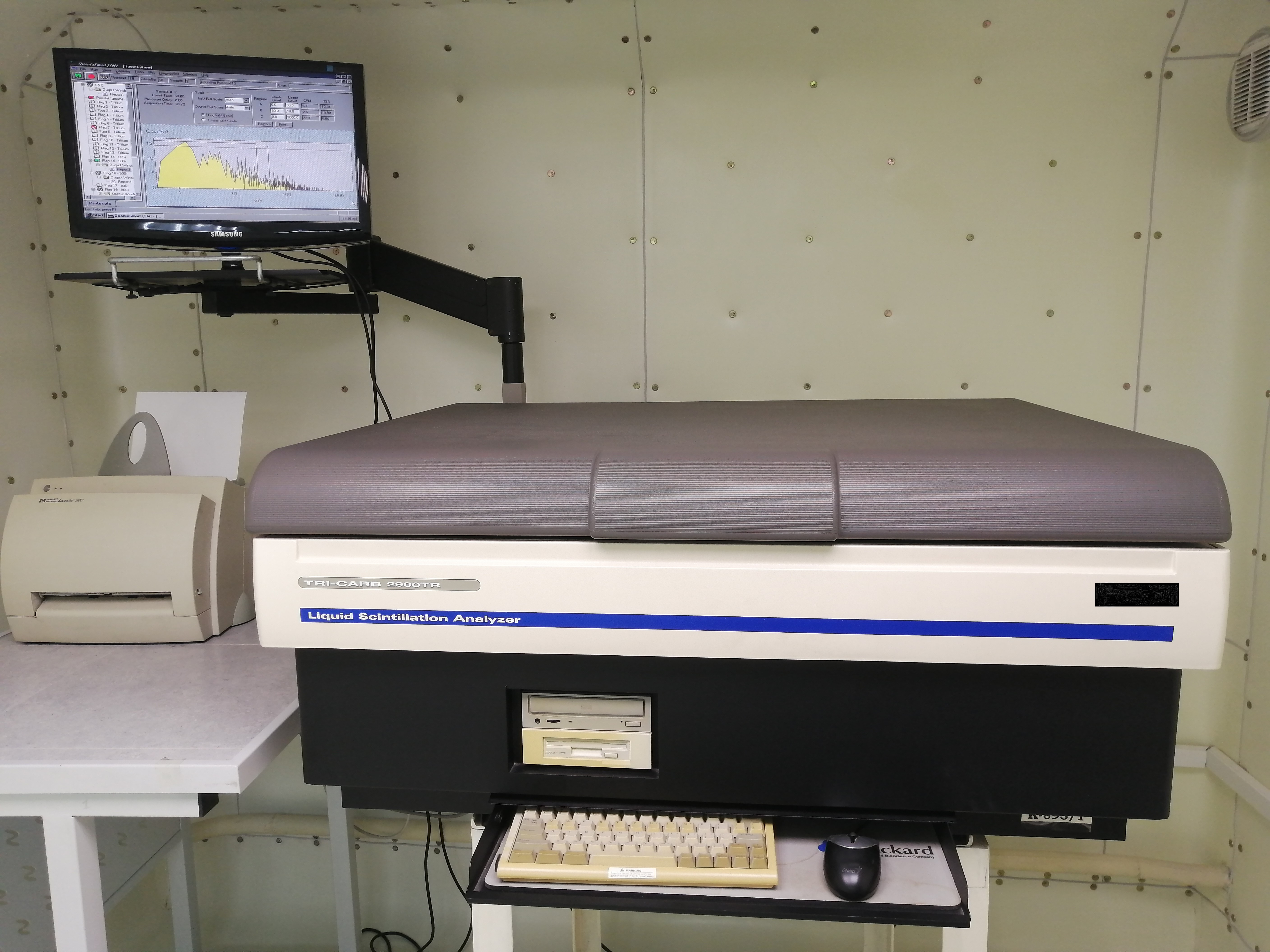


**Fig 10. Liquid scintillation spectrometer ‘TRI-CARB 2900 TR’**
